# Impact of hyperglycemia on cystathionine-γ-lyase expression during resuscitated murine septic shock

**DOI:** 10.1186/s40635-017-0140-7

**Published:** 2017-06-14

**Authors:** Tamara Merz, Josef A. Vogt, Ulrich Wachter, Enrico Calzia, Csaba Szabo, Rui Wang, Peter Radermacher, Oscar McCook

**Affiliations:** 1 Institute of Anesthesiological Pathophysiology and Process Engineering, University Medical School, Helmholtzstrasse 8-1, 89081 Ulm, Germany; 2 Department of Anesthesiology, University Hospital, Ulm, Germany; 3 Department of Anesthesiology, University of Texas Medical Branch, Galveston, TX USA; 4 Department of Biology, Laurentian University, Sudbury, ON Canada

**Keywords:** Glucose oxidation, Gluconeogenesis, Peroxisome proliferator-activated receptor gamma coactivator 1-alpha, Mitochondrial function, Lipid droplet formation

## Abstract

**Background:**

Cystathionine-γ-lyase (CSE) was shown to have a regulatory role in glucose metabolism. Circulatory shock can induce metabolic stress, thereby leading to hyperglycemia and mitochondrial dysfunction. In vitro data suggest an effect of high glucose on CSE expression. Therefore, the aim of this study was to investigate the effects of hyperglycemia on CSE expression in resuscitated murine septic shock.

**Methods:**

Normo- (80–150 mg/dl) and hyperglycemic (>200 mg/dl) male C57/BL6J mice (*n* = 5-6 per group) underwent cecal ligation and puncture (CLP)-induced polymicrobial sepsis or sham procedure (*n* = 6 per group) and, 15 h afterwards, were anesthetized again, surgically instrumented and received intensive care treatment, including antibiotics, lung protective mechanical ventilation, circulatory support, and intravenous (i.v.) glucose infusion (50% as stable-isotope labeled 1,2,3,4,5,6–^13^C_6_ glucose). Blood and breath gas were sampled hourly to quantify parameters of glucose metabolism. 5 h later, mice were sacrificed and organs were harvested. The liver mitochondrial respiratory activity was determined via high resolution respirometry; CSE, peroxisome proliferator-activated receptor gamma coactivator 1-alpha (PGC1α), and adipocyte differentiation-related protein (ADRP) expression was immunohistochemically investigated.

**Results:**

In sepsis combined with hyperglycemia the least CSE and PGC1α expression could be detected, along with reduced mitochondrial respiratory activity, and enhanced ADRP expression, a marker of lipid droplet formation, in the liver. A novel in vivo finding is the CSE translocation from the cytosol to the nucleus triggered by metabolic stress.

**Conclusions:**

A relationship between CSE and glucose metabolism was established, which, when dysregulated, may contribute to fatty liver disease and hepatic steatosis.

## Background

Recently, the gasotransmitter hydrogen sulfide (H_2_S) was shown to play a role in the regulation of glucose metabolism [1]; by enhancing glucose-generating processes and by inhibiting glucose-consuming processes, it ultimately increases glucose availability. In the liver, the central organ for glucose homeostasis, high H_2_S levels can be detected [2], mainly attributed to a high expression and activity of cystathionine-γ-lyase (CSE) [3]. In fact, in CSE knock-out mice, reduced gluconeogenesis was observed, which could be restored by exogenous supplementation of H_2_S [4]. In turn, hyperglycemia in vitro caused downregulation of CSE expression and reduction of H_2_S formation [5–7], suggesting a regulatory loop between glucose metabolism and CSE expression. Hyperglycemia is a significant factor in sepsis-induced metabolic stress and critical illness [8]. “Critical illness-associated hyperglycemia” (CIAH, [9]) in response to an acute stress is a common feature in intensive care patients [8, 10] and has actually been found to be the most frequent cause of hyperglycemia in all the critically ill [9]. Hyperglycemia leads to mitochondrial dysfunction [11] resulting from enhanced oxidative stress [12]. All the aforementioned players can contribute to the development of hepatic steatosis [13]. In fact, evidence of mitochondrial injury was associated with increased hepatocyte vacuolization in patients and murine models of sepsis [14–16]. The genetic deletion of CSE led to hepatic cholesterol accumulation and promotes the development of fatty liver [17]. Consequently, maintenance of endogenous H_2_S production might attenuate sepsis-induced metabolic alterations; due to its effects on glucose utilization, its protective effect on mitochondrial function [18], and the potential of limiting the development of liver steatosis [1]. CIAH might contribute to these alterations and interfere with CSE expression. Therefore, in a murine, resuscitated cecal ligation and puncture-induced septic shock, we tested the hypothesis whether a transient hyperglycemia in vivo would downregulate CSE expression, affect mitochondrial function, and increase the susceptibility of developing hepatic steatosis. The data presented are a post hoc analysis of material available from a previous study [11].

## Methods

All animal experiments were performed with the approval of the University Animal Care Committee and of the federal authorities for animal research of the Regierungspräsidium Tübingen, Baden-Württemberg and with adherence to the National Institutes of Health Guidelines on the Use of Laboratory Animals. We used the available liver tissue from a previous study [11] of normo- and hyperglycemic septic male C57BL6/J mice (body weight 23–29 g, age 10–16 weeks) and so far unpublished sham groups studied simultaneously.

Since mice, when critically ill, become hypoglycemic instead of hyperglycemic, due to their different metabolic phenotype [19–21], in the original study all animals got an exogenous infusion of glucose. The blood glucose values of the animals were targeted to normo- (target glycemia 100+/−20 mg/dl) or hyperglycemic (target glycemia 180+/−50 mg/dl). In this post hoc experiment, we stratified animals based on the literature to be able to dissect the in vivo relationship of CSE and glucose. Normal values reported in the literature for blood glucose in mice are highly variable and depend on the mouse strain, age, and sex [22]. For male C57/BL6J mice, they range between 80 and 150 mg/dl [22, 23]. The methods used for acquisition of the blood sample and determination of glucose affect the assessed values [24]. Therefore, we used the lowest value considered as hyperglycemic in the literature [25, 26], i.e., 200 mg/dl, to stratify animals according to high (>200 mg/dl; *n* = 6 for septic, *n* = 5 for sham) and normal (80–150 mg/dl; *n* = 6 per group) endpoint glycemia.

The anesthesia, cecal ligation and puncture (CLP) procedure, and surgical instrumentation, all have been described in detail previously [11]. Mice were anesthetized with sevoflurane and received subcutaneous (s.c.) buprenorphine together with acetated Ringer’s solution containing glucose (4 or 12 mg/g for normoglycemic and hyperglycemic animals). A midline laparotomy was performed to identify and ligate the cecum followed by a single puncture (18-gauge needle). After squeezing to expel stool, the cecum was returned into the abdominal cavity. Sham animals were treated the same, with the exception of cecal puncture. Postoperatively, water and food were provided ad libitum. After 6 h, mice received a second s.c. injection including buprenorphine, acetated Ringer’s solution containing glucose as described above together with ceftriaxone and clindamycin (each with 30 μg/g). 15 h post CLP, mice were anesthetized with sevoflurane followed by intraperitoneal (i.p.) ketamine (120 μg/g), midazolam (125 μg/g), and fentanyl (0.25 μg/g). After placement of the animal on the procedure bench equipped with a heating pad and a lamp, a rectal temperature probe was inserted. The anterior neck was incised to expose the trachea, the right internal jugular vein, and the right carotid artery. The trachea was intubated, and the lungs were mechanically ventilated with a pressure-controlled, lung-protective ventilation strategy using a small animal ventilator (FlexiVentTM, Scireq®, Montreal, Canada). After a lung recruitment maneuver, respirator settings were FiO_2_ 0.5, tidal volume from 6 to 8 μl/g (titrated to maintain arterial pCO_2_ at 30 to 40 mmHg), respiratory rate 160 breaths/min, inspiratory/expiratory time ratio 1:2, and PEEP 5 cmH_2_O. Catheters were inserted into the jugular vein, the carotid artery, and the bladder; a perivascular flow probe was placed around the portal vein in the liver organ bed. Microvascular perfusion, capillary hemoglobin concentration, and oxygen saturation were determined simultaneously every hour by a combined laser Doppler flowmetry and remission spectroscopy technique, as also described previously [19, 27]. Anesthesia was maintained with continuous i.v. ketamine, fentanyl, and midazolam, titrated to reach deep sedation and analgesia as documented by complete tolerance against noxious stimuli. Normotensive (i.e., mean arterial pressure (MAP) ≥ 55 mmHg) hemodynamics were maintained by hydroxyethylstarch infusion (20 μL/(g · h)) in a balanced electrolyte solution (Tetraspan 6%, Braun, Melsungen, Germany), together with continuous i.v. norepinephrine as needed to reach the target MAP of ≥ 55 mmHg. The targeted blood pressure in this established model [28] was chosen according to the normal values reported for mechanically ventilated C57/BL6 mice, when comparable anesthesia regimens were administered via continuous infusion [29, 30]. Septic animals were infused with 1 or 2 mg/(g∙h) (additional sham animals received 1 or 3 mg/(g∙h)) continuous i.v. glucose to achieve normo- (target glycemia 100+/−20 mg/dl) or hyperglycemic (target glycemia 180+/−50 mg/dl) conditions, respectively. At the end of the experiment, animals were sacrificed through exsanguination.

### Evaluation of glucose metabolism

Glucose metabolism was evaluated as described previously [11, 27]. 50% of i.v. glucose was given as stable, non-radioactive isotope-labeled 1,2,3,4,5,6-^13^C_6_-glucose. For the measurement of blood glucose concentrations and ^13^C_6_-glucose tracer enrichment, plasma samples were spiked with 6,6-^2^H_2_-glucose for concentration determination, with an amount targeted to achieve a ^2^H_2_-tracer mole fraction of 50%. The spiked samples were derivatized with N-methyl-bis (trifluoroacetamide) (MBTFA, abcr, Karlsruhe, Germany) to obtain the trifluoroacetyl-glucose derivative. The latter was analyzed by gas chromatography/mass spectrometry (GC/MS) under electron impact determination, and the signals at (m/z) 319, 321, and 325 were recorded for the ^2^H_2_- and ^13^C_6_-tracer mole fraction determination. Expiratory gas, 1 ml, was intermittently collected as an aliquot from 60 to 80 respiratory cycles from the expiratory branch to determine both expiratory CO_2_ concentration and ^13^CO_2_ tracer enrichment using GC/MS (masses m/z 44 and m/z 45). CO_2_ production rates (VCO_2_) were calculated as the product of tidal volume, respiratory rate, and CO_2_ concentration. During steady-state conditions, the glucose rate of appearance (Ra) was derived from the arterial plasma isotope enrichment, while glucose oxidation can be determined from ^13^CO_2_ tracer enrichment in breath gas samples.

### Mitochondrial respiration

Mitochondrial respiratory activity was determined via high-resolution respirometry with a Clark-electrode-based system (Oxygraph 2k, OROBOROS Instruments Corp., Innsbruck, Austria) as also described previously [27]. Post-mortem liver samples were collected in custodiol, mechanically homogenized in respiration medium (MIR05; 0.5 mM EGTA, 3 mM MgCl_2_ · 6H_2_O, 60 mM Lactobionic acid, 20 mM Taurine, 10 mM KH_2_PO_4_, 20 mM HEPES, 110 mM Sucrose, and 1 g/l bovine serum albumin), and 2 mg of tissue were added to the Oxygraph chamber. By the addition of a defined sequence of substrates and inhibitors, various states of mitochondrial function could be assessed. Complex I activity was determined after the addition of 10 mM pyruvate, glutamate and 5 mM malate, and ADP. 10 μM cytochrome c was added to check for mitochondrial integrity. Maximum oxidative phosphorylation (max OxPhos) was evaluated after subsequent addition of 0.5 mM octanoyl-carnitine and 10 mM succinate, leak compensation was assessed after inhibition of the ATP-synthase by 5 μM oligomycin, followed by stepwise titration of the uncoupling agent Carbonyl cyanide-*4*-(trifluoromethoxy) phenylhydrazone (FCCP, final concentration 1.5 μM) to reach maximum respiratory activity of the electron transfer system in the uncoupled state (ETS). The activity of complex II could only be determined in the uncoupled state by the addition of 0.5 μM rotenone, an inhibitor of complex I. The measurement was finished after the addition of 5 μM of the complex III inhibitor antimycin A.

### Immunohistochemistry

Identical liver biopsies were sampled immediately post mortem and were fixed in formalin for a defined period of time, dehydrated and embedded in paraffin. Immunohistochemistry (IHC) was perfomed on 3-μm sections that were deparaffinized in xylene and rehydrated in graded mixtures of ethanol to deionized water. Heat-induced antigen retrieval was performed by microwaving in citrate buffer pH 6.0, followed by blocking with 10% normal goat or donkey serum, depending on the source of the secondary antibody, as described previously [31, 32]. The following primary antibodies were used: Cystathionine-γ-lyase (anti-CSE, rabbit polyclonal, Proteintech), Adipophilin (anti ADRP, guinea pig polyclonal, Progen Biotechnik), and peroxisome proliferator-activated receptor gamma coactivator 1-alpha (anti-PGC1α, rabbit polyclonal, Novusbio). Primary antibody detection was performed with anti-rabbit or anti-guinea pig and alkaline phosphatase-conjugated secondary antibodies (Jackson Immunoresearch), was subsequently detected with Dako REAL Detection System Chromogen Red (Dako), and was counterstained with Mayers hematoxylin (Sigma). Slides were densitometrically analyzed using a Zeiss AxioImager A1 microscope, ×10 objective and the “AxioVision” image analysis software (release 4.9.1), multiple 800,000 μm^2^ fields were evaluated. Data are represented as mean densitometric sum red.

IHC for cystathionine-β-synthase (CBS) and 3-mercaptopyruvate-sulfurtransferase (MST) were performed as well; expression levels for both enzymes were negligible (data not shown). Due to the surprisingly clear and specific CSE nuclear stain with the antibody, which had never been reported before, we decided to investigate it further. In spite of the fact that it has been shown by western blot (including knock-out) to be the most specific commercially available antibody [33], we requested the manufacturer to provide additional support. Thus, they performed a CSE siRNA knock-down on HepG2 cells, showing the antibody to be clearly specific for CSE (https://www.ptglab.com/). Furthermore, a gene database search confirms that CSE should be found in the nucleus with the same confidence as in the cytoplasm (www.genecards.org).

### Statistical analysis

All data are presented as median (quartiles) unless otherwise stated. After exclusion of normal distribution using the Kolmogorov-Smirnov test, differences between all groups were analyzed with a one way Kruskal-Wallis analysis of variance on ranks followed by a post hoc Dunn’s test for multiple comparisons. Quantitative relations, of pooled data sets from all experimental groups, between CSE expression and glucose production rates and CSE expression and PGC1α were evaluated by measuring the Pearson’s coefficient of correlation for linear relationships. The relation between CSE and ADRP expression was determined with the Spearman’s coefficient for non-linear relationships. For the latter, data were fitted to an exponential decay function according to the general equation f (x) = a · e^−bx^.

## Results

### Physiological data

Table 1 summarizes the data on hemodynamics, acid base-status and norepinephrine requirements. Septic shock is defined as a state of acute circulatory failure with a need for vasopressor support to maintain MAP and elevated lactate (≥2 mmol/l) in spite of adequate fluid resuscitation [34]. Both CLP groups displayed these classical characteristics of septic shock, in contrast to the sham animals, that did not require norepinephrine administration, in addition to fluid resuscitation, to maintain their MAP. The CLP hyperglycemic group presented with statistically significant highest lactate levels. Lactate was also elevated in both hyperglycemic sham and normoglycemic CLP animals, whereas base excess was more negative only in the septic groups.

**Table 1:** Physiological data

	Normo. Sham (*n* = 6)	Normo. CLP (*n* = 6)	Hyper. Sham (*n* = 6)	Hyper. CLP (*n* = 5)	*p* value
NoA (ng/(g · h))	0 (0; 0)	20 (10; 30)	0 (0; 0)	30 (20; 100)^b, c^	0.001
HR (beats/min)	310 (310; 315)	481 (397; 503)^b^	420 (380; 450)^a^	479 (459; 492)^c^	0.002
MAP (mmHg)	60 (58; 66)	60 (56; 62)	69 (68; 73)	58 (57; 58)	0.026
Portal ven. flow (ml/min)	4.3 (3.4; 4.7)	6.1 (4.5; 11.0)	4.8 (3.4; 5.8)	6.2 (5.2; 7.3)	0.066
Liver μ-vasc. flow (AU)	122 (109; 127)	111 (93; 176)	132 (120; 148)	133 (105; 147)	0.767
Liver μ-vasc. O_2_ sat. (%)	61 (58; 63)	61 (58; 64)	64 (60; 66)	65 (60; 66)	0.481
BE (mmol/l)	−7.5 (−10.8; −5.1)	−12.9 (−14.4; −10.9)	−8.2 (−11.0; −7.6)	−13.1 (−13.9; −12.8)	0.067
Lactate (mmol/l)	1.1 (0.9; 1.4)	2.1 (1.8; 2.3)	2.1 (1.7; 2.5)	3.5 (3.4; 3.6)^a, b, c^	0.002
VCO_2_ (μl/(g · min))	20.5 (20.2; 20.6)	25.0 (23.2; 26.6)	30.4 (29.0; 33.1)^a^	26.4 (23.4; 38.9)	0.031
pH	7.45 (7.38; 7.47)	7.28 (7.23; 7.39)	7.35 (7.30; 7.37)	7.25 (7.16; 7.31)	0.112

All data is presented as median (interquartile range). Kruskal-Wallis analysis of variance on ranks with post hoc Dunn’s test for multiple comparisons

*Normo* normoglycemia, *Hyper* hyperglycemia, *NoA* norepinephrine infusion rate, *HR* heart rate, *MAP* mean arterial pressure, *μ-vasc.* microvascular, *BE* base excess, *VCO*_*2*_ CO_2_ production

^a^Significant compared to the respective normoglycemic group

^b^Significant to respective sham group

^c^Significant to normoglycemic sham group

### Glucose metabolism

**Fig. 1:**
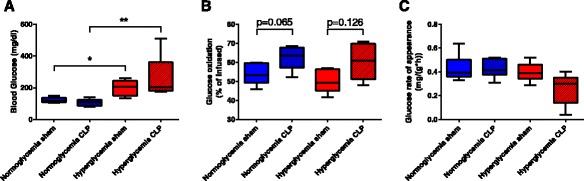
Glucose utilization during ICU (intensive care unit) treatment. **a** Blood glucose levels at 5 h of ICU, ^*^ *p* ≤ 0.05, ^**^ *p* ≤ 0.01. **b** Glucose oxidation (percentage of oxidized tracer) at 4 h of ICU. **c** Glucose rate of appearance at 5 h of ICU. All data is presented as median (interquartile range). Kruskal-Wallis analysis of variance on ranks with post-hoc Dunn’s test for multiple comparisons

Data on glucose metabolism are depicted in Fig. 1. Animals from the hyperglycemic groups had higher blood glucose levels in comparison to the normoglycemic groups (*p* = 0.001). Glucose production was lowest in the hyperglycemic septic animals, but this difference did not reach statistical significance (*p* = 0.069). There were no differences in the glucose production rates between the other experimental groups. Whole body CO_2_ production was significantly elevated in the hyperglycemia sham arm in comparison to normoglycemia (*p* = 0.031, see also Table 1). Glucose oxidation was higher in the septic groups than in the respective sham animals (*p* = 0.038), but did not differ between normo- and hyperglycemia.

### Mitochondrial function

**Fig. 2:**
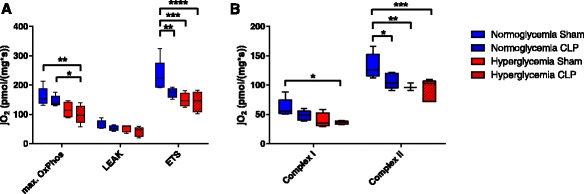
Mitochondrial respiratory activity. jO_2_ oxygen flux. **a** Maximum respiratory activity in the coupled (max OxPhos), leak (LEAK), and uncoupled state (ETS). ^*^ *p* ≤ 0.05, ^**^ *p* ≤ 0.01, ^***^ *p* ≤ 0.001, ^****^ *p* = ≤ 0.0001. **b** Maximum respiratory activity in the coupled state depending on complex I activity (*Complex I*) and maximum respiratory activity in the uncoupled state with inhibition of complex I (*Complex II*). ^*^ *p* ≤ 0.05, ^**^ *p* ≤ 0.01, ^***^ *p* ≤ 0.001. All data is presented as median (interquartile range). Kruskal-Wallis analysis of variance on ranks with post hoc Dunn’s test for multiple comparisons

Oxygen consumption in various respiratory states of liver mitochondria is given in Fig. 2. Maximum oxidative phosphorylation was significantly reduced in the hyperglycemic septic animals in comparison to the normoglycemic groups (*p* = 0.024). Leak compensation flux did not differ between the groups, whereas the maximum respiratory activity was significantly reduced by hyperglycemia as well as sepsis (*p* = 0.009). Reduced mitochondrial activity was mostly associated to impaired function of complex II (*p* = 0.008), activity of complex I was only significantly reduced in the hyperglycemic septic animals in comparison to normoglycemia sham (*p* = 0.016).

### Protein expression

**Fig. 3:**
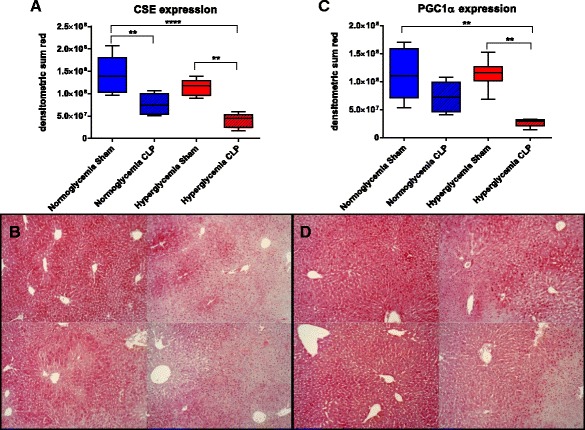
Protein immunohistochemistry in the liver. **a** CSE expression, ^**^ *p* ≤ 0.01, ^****^ *p* ≤ 0.0001. **b** Exemplary pictures for CSE expression (10×, *top left* normo sham, *top right* normo CLP, *bottom left* hyper sham, *bottom right* hyper CLP) **c** PGC1α expression, ^**^ *p* ≤ 0.01 **d** exemplary pictures for PGC1α expression (10×, *top left* normo sham, *top right* normo CLP, *bottom left* hyper sham, *bottom right* hyper CLP). All data is presented as median (interquartile range). Kruskal-Wallis analysis of variance on ranks with post hoc Dunn’s test for multiple comparisons

**Fig. 4:**
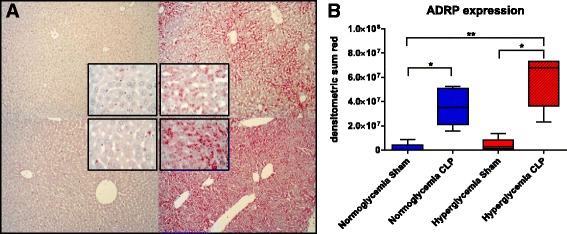
Lipid droplet formation in the liver. **a** Exemplary pictures for ADRP immunohistochemistry (10×, insets 40×, *top left* normo sham, *top right* normo CLP, *bottom left* hyper sham, *bottom right* hyper CLP) **b** ADRP expression, ^*^ *p* ≤ 0.05, ^**^ *p* ≤ 0.01. All data is presented as median (interquartile range). Kruskal-Wallis analysis of variance on ranks with post hoc Dunn’s test for multiple comparisons

**Fig. 5:**
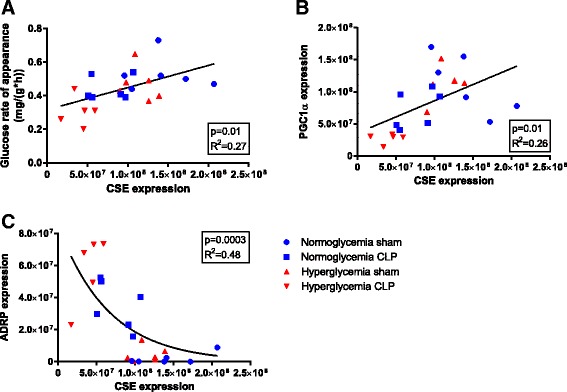
Glucose rate of appearance (**a**), peroxisome proliferator-activated receptor gamma coactivator 1-alpha (PGC1α) (**b**) and adipocyte differentiation-related protein (ADRP) (**c**) plotted as a function of cystathionine-γ-lyase (CSE) expression, pooled data from all experimental groups. *R* ^2^ is the Pearson’s coefficient for linear, and Spearman’s coefficient for non-linear relationships

Representative immunohistochemistry images and the respective quantitative analysis for CSE, PGC1α, and ADRP expression in the liver are shown in Figs. 3 and 4. Liver CSE expression in the sham groups was highest in the normoglycemic animals, and slightly reduced in the hyperglycemic arm, though not statistically significant. Induction of sepsis led to a significant reduction of CSE expression for both normo- and hyperglycemic animals; the lowest CSE expression was observed in the hyperglycemic septic arm (*p* = 0.001). The loss of CSE in the cytosol, particularly in regions of injury, was concomitant with an apparent translocation to the nucleus. Higher CSE expression correlated with higher glucose production rates (*p* = 0.011, see Fig. 5a) when analyzing pooled data from all experimental groups.

The expression of the mitochondrial biogenic factor peroxisome proliferator-activated receptor gamma coactivator 1-alpha (PGC1α) showed a comparable pattern and did also correlate with CSE expression (*p* = 0.012) (see Fig. 5b, pooled data). PGC1α was not affected by hyperglycemia alone, but sepsis led to a slight reduction, and the combination of both sepsis and hyperglycemia led to significantly and drastically reduced PGC1α levels (*p* = 0.002).

As shown in Fig. 4, hyperglycemia alone did not significantly induce lipid droplet formation; in contrast, ADRP expression was significantly increased in sepsis (*p* = 0.002). The hyperglycemic CLP animals had the highest ADRP levels. In particular the reduction of CSE and PGC1α staining around the central vein was concomitant with increased ADRP expression in the same region. Pooled data from all experimental groups revealed a negative exponential correlation between CSE and ADRP expression (*p* = 0.0003) (see Fig. 5c).

## Discussion

This study was to investigate whether high glucose would affect CSE expression in the liver and thereby lead to hepatic metabolic disorders under normal and septic conditions. The main findings were (i) a significant downregulation of CSE in the liver of septic mice with hyperglycemia along with (ii) apparent nuclear translocation of the CSE protein under stress conditions (iii) reduced glucose production, (iv) reduction of mitochondrial respiratory activity, and (v) a significant downregulation of PGC1α, ultimately being associated with (vi) fat accumulation in the liver.

Hyperglycemia led to reduced mitochondrial activity in this study, characterized by decreased maximum respiration in the uncoupled state, which was due to complex II. Interestingly, the attenuated mitochondrial function reflected in complex II in the hyperglycemic sham arm, was similar to that observed in normoglycemic CLP animals. In hyperglycemic sham animals, we only observed a small downregulation of CSE by high glucose, albeit coinciding with elevated lactate levels, which did not reach statistical significance. Acute hyperglycemia, apparently due to the short duration of the glucose administration, had only minimal effects, in contrast to the effects shown for chronic hyperglycemia [35]. The hyperglycemic sham arm presented with the highest overall metabolic rate, i.e., CO_2_ production (VCO_2_), indicating that substrates were being cleared rather than accumulated, thus helping to explain the low levels of ADRP expression in the livers. In contrast to our study, all previous experiments that have shown a downregulation of CSE by high glucose have been performed in isolated cell systems in vitro [6, 7, 36].

The septic hyperglycemic group presented with a significant downregulation of liver CSE, which in turn coincided with compromised gluconeogenesis as evidenced through the lowest glucose rate of appearance, confirming the finding of Untereiner et al. [4]. PGC1α expression, which was shown to be regulated by H_2_S in the induction of gluconeogenesis [4], was also significantly reduced in the hyperglycemic septic arm. The reduced mitochondrial respiratory activity in this group was characterized by a decrease in the maximum activity in both the coupled (maximum oxidative phosphorylation, OxPhos) and uncoupled (maximum capacity of the electron transfer system) state. Both complex I and II function was reduced as well. This might also be related to the marked downregulation of PGC1α, which is, as a mitobiogenic factor and regulator of OxPhos proteins, of critical importance for maintenance of mitochondrial function [37–41]. Reduced activity of mitochondrial complex I was associated with decreased glutathione levels [42] and was prevalent in septic patients [43], as well as in CLP-induced rodent sepsis [44]. This may not only be due to transcriptional regulation but also due to post-translational regulation. In this context, reduced activity of mitochondrial complex I activity is likely also caused by S-nitrosation of critical sulfhydryl groups (as shown to occur when high fluxes of NO are produced over prolonged periods of time [45]).

The reduction of PGC1α may not only lead to impaired function but to a total decrease in the number of mitochondria [39]. This resulting state of reduced mitochondrial respiration is a sign of injury, which has also been confirmed in experimental CLP models [14]. In the mouse liver, the loss of PGC1α and reduced mitochondrial activity have been reported to manifest in enhanced vacuole formation and fatty liver disease [39]. This is also reported for septic patients: morphologically, post mortem liver sections from these patients show increased hepatic vacuolization [16]. Fatty liver disease is diagnosed histologically by the presence of fat droplets through ADRP immunohistochemistry [46, 47]. Accordingly, the strongest induction of lipid droplet formation was observed in the septic hyperglycemic group, where the ADRP expression, as a marker of liver injury, was significantly elevated. Finally, the accumulation of ADRP positive fat droplets was accompanied by a loss of CSE and PGC1α expression in the injured liver.

These findings were corroborated by the physiological results, which demonstrated that the hyperglycemic septic animals had the highest norepinephrine requirements to maintain the targeted MAP, i.e., 1.5-fold higher than CLP in the presence of normoglycemia. Lactate, both documenting a more severe septic shock as well as reduced hepatic gluconeogenesis [48], was significantly elevated in the septic hyperglycemic arm. Notably, the sham hyperglycemic group presented with similarly elevated lactate levels as the normoglycemic CLP group, suggest that there was a metabolic imbalance induced by hyperglycemia. Both septic groups had more severe metabolic acidosis than the sham groups, thus indicating that indeed the elevated lactate in the hyperglycemic sham group was to a large extent a sign of derangements in glucose metabolism.

These results are consistent with the findings reported above that the septic hyperglycemic group presented with the lowest glucose rate of appearance. This group was further characterized by a modestly elevated glucose oxidation, which might be attributed to the administered norepinephrine [49], together with unobtrusive overall CO_2_ production. This might hint to a shift towards glucose oxidation and reduced fat utilization. Other authors refer septic shock-related liver dysfunction to reduced hepatic arterial blood flow [20]. We did not assess hepatic arterial blood flow, but in our study, neither portal vein flow (i.e. hepatic macrovascular flow) nor liver microvascular perfusion or hemoglobin O_2_ saturation differed between the experimental groups. Hence, the observed liver damage was most likely not due to malperfusion or tissue hypoxia, but may indeed, be due to the cellular inability to utilize oxygen [42], as evinced by the reduced mitochondrial activity associated with the downregulation of CSE and PGC1α.

Mice, when critically ill, become hypoglycemic instead of hyperglycemic, due to their different metabolic phenotype, which is why in our study animals received exogenous glucose administration [20, 21]. Thus, the rate of gluconeogenesis reflects the metabolic capacity of the liver [19]. The effects of sepsis on the liver reported here are all aggravated in combination with hyperglycemia. Additionally, we could observe derangements in glucose metabolism, as indicated by the reduced glucose rate of appearance. The loss of CSE might further impair hepatic metabolic activity [50], and thereby dysregulate glucose metabolism.

The IHC evaluation of the tissue not only helped identify topography of protein expression in regions affected by septic shock, but also eliminated confounding results due to contamination with occult blood products, a common occurrence in processed tissue [51]. We were not only able to observe a loss of CSE around the central veins, which was most pronounced in the septic groups, but were also able to notice a conspicuous absence of CSE expression in the endothelium of the central veins and hepatic arterioles in all groups. This is in agreement with the literature wherein CSE protein expression is reported to be evenly distributed in hepatocytes [52–54], but there is no mention of it being specifically expressed in the liver endothelium. An interesting novel finding is the presence of CSE-positive nuclei in the hepatocytes of injured mouse liver. A review of the literature of the IHC CSE localization experiments (all with different antibodies) demonstrates varying positive nuclear staining for CSE, though not specifically mentioned [52, 54–56]. In fact, it has been shown in vitro that CSE [55, 57] can translocate to the nucleus. It is speculated that CSE re-localizes to the nucleus to increase cysteine levels, a needed substrate to produce glutathione, when the demand for nuclear glutathione is increased [58, 59]. Decreased glutathione, associated with impaired mitochondrial activity, are common indicators for the severity of sepsis in patients and prognosticator of death [42]. This is reflected in our study where the reduced mitochondrial activity is present in the septic arms, which also display diminished CSE expression concomitant with nuclear CSE translocation, a reduction of PGC1α and an increase in ADRP, a marker of liver injury [46].

## Conclusions

This *post hoc* study demonstrated, for the first time, in vivo, in a clinically relevant resuscitated mouse model of septic shock, that there is a direct effect of glucose on CSE expression. Hyperglycemia in sepsis can downregulate CSE expression in the liver and thereby compromise PGC1α signaling, reduce mitochondrial activity, and promote lipid droplet formation—all early signs of liver damage, which may ultimately manifest as fatty liver disease and hepatic steatosis [17].

## Abbreviations

ADRP: Adipocyte differentiation-related protein
BE: Base excess
CBS: Cystathionine-β-synthase
CIAH: Critical illness-associated hyperglycemia
CLP: Cecal ligation and puncture
CSE: Cystationine-g-lyase
ETS: Electron transfer system
FCCP: Carbonyl cyanide-4-(trifluoromethoxy) phenylhydrazone
GC/MS: Gas chromatography/mass spectrometry
H2S: Hydrogen sulfide
HR: Heart rate
hyper: Hyperglycemia
ICU: Intensive care unit
IHC: Immunohistochemistry
MAP: Mean arterial pressure
MIR05: Respiration medium
MST: 3-mercaptopyruvate-sulfurtransferase
normo: Normoglycemia
OxPhos: Oxidative phosphorylation
PGC1α: Peroxisome proliferator-activated receptor gamma coactivator 1-alpha
μ-vasc.: Microvascular
